# Retraction Note: BICC1 drives pancreatic cancer progression by inducing VEGF-independent angiogenesis

**DOI:** 10.1038/s41392-025-02561-9

**Published:** 2026-01-02

**Authors:** Chongbiao Huang, Hui Li, Yang Xu, Chao Xu, Huizhi Sun, Zengxun Li, Yi Ge, Hongwei Wang, Tiansuo Zhao, Song Gao, Xiuchao Wang, Shengyu Yang, Peiqing Sun, Zhe Liu, Jing Liu, Antao Chang, Jihui Hao

**Affiliations:** 1 Department of Pancreatic Cancer, Tianjin Medical University Cancer Institute and Hospital, National Clinical Research Center for Cancer, Tianjin Key Laboratory of Digestive Cancer, Key Laboratory of Cancer Prevention and Therapy, Tianjin, China; 2 Department of Anorectal Surgery, The Second Hospital of Tianjin Medical University, Tianjin, China; 3 Department of Cellular and Molecular Physiology, the Pennsylvania State University College of Medicine, Hershey, PA USA; 4 Department of Cancer Biology, Wake Forest Baptist Comprehensive Cancer Center, Wake Forest Baptist Medical Center, Winston-Salem, NC USA; 5 Department of Immunology, School of Basic Medical Sciences, Tianjin Medical University, Tianjin, China

Retraction to: *Signal Transduction and Targeted Therapy* 10.1038/s41392-023-01478-5, published online 14 July 2023

The Editors have retracted this article. After publication, a number of image integrity concerns were raised. Specifically:

- Two panels in figure 4F appear to overlap representing BICC1, BICC1+LCN2ab, and BICC1, BICC1+CXCL1ab, which was subsequently corrected.
- Figure S10b BxPC-3 BICC1 SN+VEGFAab appears to overlap with figure S10c KPC BICC1 SN.
- Figure S10b AsPC-1 BICC1 SN+VEGFAab appears to overlap with figure S11b BICC1 SN LCN2ab -, BxPC-3.
- Figure S10b AsPC-1 BICC1 SN appears to overlap with figure S11b BICC1 SN LCN2ab -, AsPC-1.
- Figure S11c vector SN CXCL1ab - BxPC-3 appears to overlap with Figure S11d CFPAC-1 rhLCN2 30 min CXCL1ab -.
- Figure S4e furthest left mouse image for Pan02 shBICC1 appears to overlap with figure S12b furthest left mouse image for KPC vector.

The Editors have lost confidence in the data and conclusions of this article.

Author Shengyu Yang agrees with this retraction. All other authors did not respond to correspondence from the publisher regarding this retraction.

